# Cross-lagged analysis of social support, physical activity behavior, and family relationships among university students

**DOI:** 10.3389/fpsyg.2024.1439252

**Published:** 2024-08-16

**Authors:** Xielin Zhou, Mu Zhang, Bo Li, Shasha Ma

**Affiliations:** ^1^School of Sports Training, Chengdu Sport University, Chengdu, Sichuan, China; ^2^Information Technology Centre, Chengdu Sport University, Chengdu, Sichuan, China; ^3^School of Graduate Education, Shandong Sport University, Jinan, Shandong, China

**Keywords:** social support, physical activity behavior, family relationships, college students, cross-lagging

## Abstract

**Objective:**

To explore the causal relationship between social support, physical activity behavior, and family relationships among university students.

**Methods:**

Using the Social Support Rating Scale, the Physical Activity Behavior Self-Assessment Scale, and the Family Relationships Scale, a longitudinal follow-up survey was conducted on 412 college students in Sichuan Province at 2-month intervals in March 2024 (T1) and May 2024 (T2), to analyze the interaction mechanisms between college students' social support, physical activity behaviors, and family relationships through cross-lagging.

**Results:**

(1) There are significant gender differences in social support, physical activity behavior, and family relationships among college students. Among the cross-lagged paths found, except for the path from T1 social support to T2 family relationships (β: 0.40 > 0.21), all other cross-lagged paths are smaller for female college students compared to male college students; (2) T1 social support was able to positively predict T2 physical activity behaviors (β = 0.50, *p* < 0.001), and T1 physical activity behavior can also positively predict T2 social support (β = 0.18, *p* < 0.01), but the path value T1 social support → T2 physical activity behavior is larger than T1 physical activity behavior → T2 social support. Therefore, social support is a causal variable for physical activity behavior; (3) T1 social support positively predicts T2 family relationships (β = 0.26, *p* < 0.001); (4) T1 family relationships positively predict T2 physical activity behavior (β = 0.30, *p* < 0.001). (5) Physical activity behavior is a mediating variable between family relationships and social support, with a mediating effect size of 0.054.

**Conclusion:**

There are gender differences in social support, physical activity behavior, and family relationships among college students; there is a longitudinal causal relationship between social support, physical activity behavior, and family relationships; social support is a causal variable of physical activity behavior, and social support is also a causal variable of family relationships, and family relationships are the Social support is a causal variable for physical activity behavior, social support is also a causal variable for family relations, and family relations are a causal variable for physical activity behavior, which has a partially mediating role in family relations and social support.

## 1 Introduction

Physical activity behavior refers to the physical activities in which individuals actively participate to enhance their health, improve their motor skills, and promote good exercise habit (Garber et al., 2011). Physical exercise is essential to promoting the healthy development of an individual's body and mind. For college students, it is a crucial means of promoting their all-round development (Mandolesi et al., 2018). However, the physical fitness of college students in China is on the decline, and psychological problems are on the rise. The survey data from China Youth Network Campus Communication reveals a startling fact: a staggering 48.19% of college students exercise < 3 times a week, while a whopping 58.7% of college students exercise for no more than 30 min at a time (http://txs.youth.cn)^1^. Moreover, the Chinese Academy of Sciences and other departments have released the 2022 Survey Report on the Mental Health Status of College Students, which shows that 21.4 percent of college students are at risk of depression and are affected by pressures such as further education and employment (http://www.psych.ac.cn) (Institute of Psychology, Chinese Academy of Sciences, 2022). The current situation is worrisome since the issue of the physical and mental health of college students is related to the development of the country. In recent years, the Chinese government has issued documents such as the National Physical Fitness Standard for Students, Basic Standard for Physical Education in Higher Education Schools, Measures for Monitoring and Evaluating Students' Physical Fitness and Evaluation, and Guidelines for Mental Health Education of Students in Higher Education Schools, aiming to promote college students' physical exercise, improve their psychological problems, and promote their all-round development. Several studies have indicated that the physical activity behaviors of college students are influenced by several factors, including family, peers, academic pressure, and gender (Garcia et al., 2016), among which, the family relationship plays a pivotal role in the formation of familial education and values. In addition, studies have found that the family and parents are the primary determinants of children's engagement in physical activity (Zhu et al., 2003). Meanwhile, social support, especially the family and peer support influences college students' physical activity behavior (Bandura, 2004). However, there is a lack of discussion in the academic community on the mechanism of influence between college students' physical activity behavior, social support, and family relationships, and some of the existing studies are mostly discussed in a cross-sectional way, which cannot determine the trend and pattern of individual changes over time and the dynamic relationship between variables. Hence, what is the longitudinal relationship between college students' physical activity behavior and family relationships and social support? This question has not been adequately argued so far. Based on this, the present study attempts to analyze the causal links between social support, physical activity behavior, and family relationships among college students from a longitudinal tracking perspective, which can enrich the research on the development of physical and mental health of college students and provide some references for the active participation of college students in physical activity and the improvement of physical activity behavior.

### 1.1 Social support and physical activity behavior

Social support is defined as an interactive relationship that encompasses providing emotional or material assistance between individuals, which is a crucial factor in the growth and maintenance of human physical and mental wellbeing (Feeney and Collins, 2015). Social support can provide individuals with material and spiritual help (Song et al., 2024), which is mainly manifested in enhancing the individual's ability to resist stress, providing positive emotions (Sun et al., 2023), and decreasing the probability of depressive symptoms, thus promoting the physical and mental health development of human beings (Kaitlin and Erin, 2018).

Physical activity behavior refers to physical activities in which individuals actively participate to enhance their health, improve their motor skills, and promote good exercise behavior (Garber et al., 2011). Studies have shown that the frequency of participation in physical activity is negatively associated with the risk of developing depression (Schuch et al., 2018). This is attributed to engaging in healthy physical activities conducive to fostering positive emotions, enhancing moral values, and shaping desirable characters among participants. Additionally, it serves as a distinct medium for promoting the development of socio-emotional proficiency (Zhang, 2021). At the same time, the higher the level of physical activity behaviors, the greater the subjective support and support utilization that can be gained, and the fewer the negative emotions that arise (Smith et al., 2017). The preceding studies indicate that physical activity behavior and social support commonly influence the reduction of depressive symptoms and negative emotions. To ascertain whether physical activity behavior can exert a direct effect on social support, some studies have demonstrated that among college students, physical activity can enhance the level of social support and psychological wellbeing of individuals (Chen, 2001; Cui et al., 2002; Fang and Zhao, 2005). Therefore, this paper proposes Hypothesis 1a: Physical activity behavior positively predicts social support across time in the college student population.

College is a transitional stage between adolescence and adulthood, and the development of physical activity behavior at this stage will have an important impact on future outcomes (Ji et al., 2022). A review of existing research indicates that the level of physical activity among college students in China is relatively low. This is evidenced by the low frequency of participation in physical activity, substandard exercise volume and intensity, and poor physical fitness (Wang et al., 2009). It has been shown that support through peer relationships is particularly important during physical activity in adolescents, mainly in the form of a greater impact of group exercise than solitary exercise on individual exercise persistence and development, i.e., the social support that individuals receive through group exercise during adolescence contributes to increased persistence in exercise, which in turn influences life and habits in college (Dollman, 2018; Ma et al., 2023). At the same time, the study further pointed out that at the university level, the occurrence of physical activity behavior is influenced by subjective and objective factors, including the individual's interest in sports, the importance of sports, sports habits, etc., and objective factors, including social factors, school factors, and family factors (Huang and Zhang, 2020). Furthermore, social support among social factors, especially family and peer support influence college students' physical activity behavior (Bandura, 2004). Based on this, this paper proposes hypothesis 1b: For the college student population, social support positively predicts physical activity behavior over time.

### 1.2 Family relationships and physical activity behavior

Physical activity behavior is influenced by various aspects such as individual, family, and society (Pan et al., 2022). Previous studies have shown that the family is the initial place to cultivate individuals' physical activity behavior, and family relationships have an important influence on family members' physical activity behaviors and habits, and a good family atmosphere and family relationships can increase the frequency of individuals' participation in physical activity (Wang et al., 2016), and at the same time, familial relationships that are characterized by a high degree of closeness are more conducive to the formation of positive exercise habits among family members (Wang, 2019). It has also been suggested that the level of parental support can positively influence the attitude of children aged 10–22 toward physical activity, and parental sports participation can also promote children's sports participation (Rhodes et al., 2020). Therefore, this study proposes Hypothesis 2a: Family relationships can positively predict physical activity behavior.

The term “family sports” is used to describe a specific type of sports activity that originated from the family unit. This concept is based on the idea of family members engaging in physical exercise activities together, whether at home or in the surrounding environment (Pan et al., 2022). Family sports have a significant impact on the evolution of family relationships, and exercise activists among family members can drive other members to participate in physical exercise (Wang et al., 2016). However, considering that many Chinese college students reside primarily on campus, it is noteworthy that the frequency of exercising with their family members during the school term is significantly diminished (He and Yang, 2014). The development of family sports activities faces difficulties. In addition, related studies have emphasized that the physical activity of Chinese college students during school is more casual (Sánchez-Herrera et al., 2022), and their motivation for participating in physical activity during school is mainly to improve physical health, recreation, and weight loss (Sánchez-Herrera et al., 2022), with no strong correlation with the development of family relationships. Therefore, the influence of physical activity behaviors on family relationships in the college student population is constrained by time and space. Based on this, this study proposes Hypothesis 2b: Physical activity behavior does not positively predict family relationships in the college student population.

### 1.3 Family relationships and social support

Family has an important influence on the formation of individual character and psychological development (Ahlberg et al., 2020), and as the public's attention to the psychological development of individuals is gradually increasing, family construction is receiving more and more attention from all walks of life (Stark et al., 2021). Family relationship is an important part of family building, which refers to the contact and communication between family members, including intergenerational (vertical) and peer (parallel) relationships (Melton et al., 2022). The theory of family functioning holds that family intimacy and adaptability can reflect family relationships to a certain extent (Olson et al., 1979). In family relationships, good parent-child (intergenerational) relationships can cultivate children's outlook on life, values, and worldview, and promote the development of social interpersonal relationships (Gruijters, 2017). In addition, previous studies have shown that interpersonal relationships are closely related to social support, which is manifested in the fact that the higher the social support, the lower the interpersonal relationship distress (Cohen and McKay, 2020).

In the meantime, some studies have shown that social support is closely related to family relationships (Michel et al., 2010), and situational factors (e.g., family relationships) affect social support (Hartley and Coffee, 2022). The study further concluded that social support mainly comes from family, lovers, friends, etc. (Cobb, 1976). In the college student population, social support is influenced by several factors, including individual psychology, the school environment, societal norms, and familial dynamics. Family relationships, specifically those related to intimacy and adaptability, have been found to have a consistent and positive correlation with social support (Zhao et al., 2011; He et al., 2018; Li and Jiang, 2023), therefore, this study proposes Hypothesis 3a: College students' family relationships predict social support.

Family conflict has been conceptualized as a poor family relationship. Some studies have demonstrated that social support can mitigate family conflict and, as a consequence, enhance family relationships (Selvarajan et al., 2016). Meanwhile, the family capital theory suggests that: family relationships, including parental relationships, exert a profound influence on the development of children. Furthermore, social support plays a crucial role in shaping these relationships and fostering a sense of purpose and meaning in life (Wei et al., 2021). Research further shows that social support affects family parent-child relationships to a certain extent (Nan et al., 2015). Therefore, this study proposes Hypothesis 3b: Social support positively predicts family relationships.

**Figure 1:**
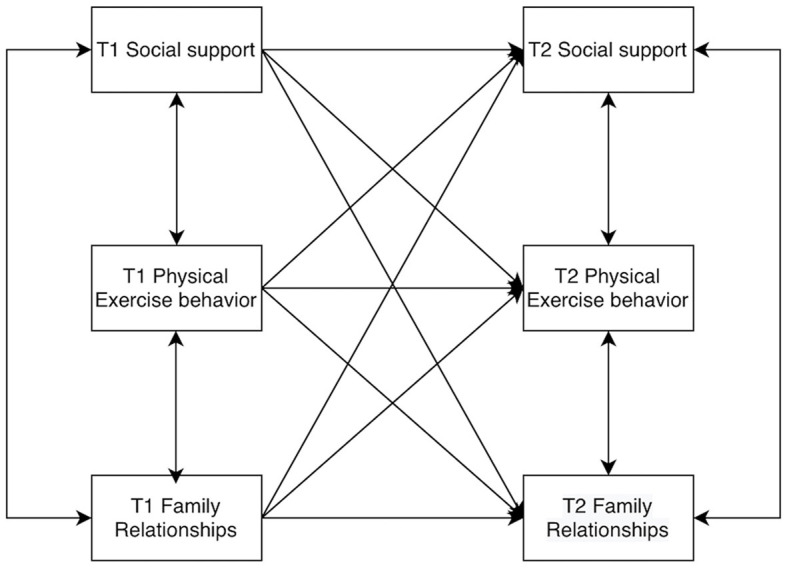
Cross-lagged structural model diagram.

Furthermore, by examining the interrelationships between family relationships and physical activity behavior, as well as between physical activity behavior and social support, it can be posited that college students' physical activity behavior may play a longitudinal and stable correlation role in family relationships and social support. Consequently, the present study proposes Hypothesis 4: When college students' family relationships affect social support, physical activity behavior may have a mediating role. Assume that the model is as shown in Figure 1.

## 2 Participants

The present study adopted the principle of convenience sampling, and the subjects were college students from Universities X and Y in S City, Sichuan Province, who were followed longitudinally for a period of 8 weeks from March to May, considering that college students have a certain degree of concentration of physical activity behavior during their school years. After informing the counselors of the introduction of the questionnaire and entrusting them to explain the purpose of the study to the subjects and to assure them that the information on the questionnaire would be kept strictly confidential, the questionnaires were answered and collected by the subjects within a specified period. The study was divided into two time points, a total of 461 questionnaires were distributed at each time point. Measurements at time point T1 were conducted offline from 5–7 March 2024, and based on the screening principles of “missing information” and “reverse questioning”, a total of 23 invalid questionnaires were rejected, and 451 valid data sets were collected, with a valid recovery rate of 95.01%”; Due to various reasons, including illness and other objective factors, some subjects were unable to return to school in time for the T2 measurement (May 6–8, 2024). Consequently, the final recovery of valid questionnaires was 432, with an effective recovery rate of 93.7%. The final valid sample size was 412 individuals who completed the questionnaire twice and had their student ID numbers matched in the correct order. The effective rate was 89.3%. Among them, 201 male college students accounted for 48.8% of the total, and 211 female college students accounted for 51.2%, Mean age (19.94 ± 1.43) years.

## 3 Method

### 3.1 Social support rating scale

The Social Support Rating Scale developed by Ye and Dai (2008) was used, which is based on Xiao (1994) theoretical model of social support, and includes three factors, subjective support, objective support, and support utilization. A total of 17 questions were included, with a 5-point Likert scale (1 = completely non-compliant, 5 = compliant completely). The higher the score on the scale, the higher the level of social support. In this study, the Cronbach α coefficients at the T1 and T2 time points were 0.873 and 0.876, respectively.

### 3.2 Physical activity behavior self-rating scale

The Self-Assessment Scale of Extracurricular Physical Activity Behavior (Zhao et al., 2015) compiled by Zhao and Fa and Li, which applies to current college students, was used, which includes three dimensions: psychological mechanisms, individual characteristics, and social environment. The scale has a total of 50 questions, due to the considerable number of questions, this study employed a method of equal censure for the three dimensions, ultimately utilizing 18 of them. Using a 5-point Likert scale (1 = not at all compliant, 5 = fully compliant), the higher the score on the scale, the higher the level of physical activity behavior. In the present study, the Cronbach alpha coefficients for the T1 and T2 time points were 0.879 and 0.892, respectively.

### 3.3 Family relationships scale

The Family Intimacy and Adaptability Scale (Fei et al., 1991) was chosen to evaluate the family relationships of college students. It contains 16 question items including the dimensions of intimacy and adaptability and is scored on a 5-point Likert scale (1 = completely non-compliant, 5 = compliant), with the 13–16 questions being reverse-scored. In the current study, the Cronbach alpha coefficients for the T1 and T2 time points were 0.914 and 0.920, respectively.

### 3.4 Statistical methods

Using SPSS27.0 and AMOS26.0, the common method bias test, descriptive statistics, correlation analysis, independent samples *t-*test, and ANOVA were performed sequentially through SPSS27.0; the cross-lagged model was constructed, and the autoregressive coefficients and cross-lagged coefficients were examined to analyze the longitudinal relationship between the college students' social support, physical activity behaviors, and family relationships using AMOS26.0.

## 4 Results

### 4.1 Common method bias test

In this study, the common method bias was examined by Harman's one-way test (Zhou and Long, 2004). The results of T1 extracted a total of 11 factors with characteristic root >1, of which the first factor cumulatively explained 29.36% of the total variance, which was less than the 40% criterion; the results of T2 extracted a total of 13 factors with Eigen roots >1, of which the first factor cumulatively explained 23.91% of the total variance, which is also less than the critical criterion of 40%. This indicates that there is no significant common method bias in both the initial and subsequent measurements of this study.

### 4.2 Descriptive statistics and correlation analysis of college students' social support, physical activity behavior, and family relationships

In this study, descriptive statistics and correlation analysis were performed on the three variables of social support, physical activity behavior, and family relationships, and the results are shown in Table 1. At the correlation analysis, it was found that the social support score, the physical activity behavior score, and the family relationship score showed a significant positive correlation (*P* < 0.01) at both T1 and T2. A significant positive correlation was found between the social support score at the 2-time points (*P* < 0.01); a significant positive correlation was found between the physical activity behavior score at the 2-time points (*P* < 0.01); and a significant positive correlation found between the family relationship score at the 2-time points (*P* < 0.01). Significant positive correlation (*P* < 0.01) was shown between family relationship scores (Table 1).

**Table 1:** Mean, standard deviation, and correlation analysis of social support, sports lifestyle, and family relationship among university students.

	**M ±SD**	**X1**	**X2**	**Y1**	**Y2**	**Z1**	**Z2**
T1Social support	3.11 ± 0.56	1					
T2Social support	3.09 ± 0.58	0.474^**^	1				
T1Physical Exercise behavior	3.16 ± 0.62	0.777^**^	0.439^**^	1			
T2Physical Exercise behavior	3.16 ± 0.61	0.782^**^	0.405^**^	0.675^**^	1		
T1Family Relationships	3.21 ± 0.66	0.631^**^	0.256^**^	0.554^**^	0.682^**^	1	
T2Family Relationships	3.17 ± 0.68	0.534^**^	0.239^**^	0.458^**^	0.482^**^	0.600^**^	1

“^*^” indicates *p* < 0.05 and “^**^” indicates *p* < 0.01 (due to table space limitations, X1 = T1 social support, X2 = T2 social support, and so on).

### 4.3 Independent samples *t*-test and ANOVA for social support, physical activity behavior, and family relationships among university students

In the current study, an independent samples *t*-test was conducted on the gender of the subjects at the two-time points (as shown in Table 2), and Levine's test of equivalence of variances for gender showed that T1 social support (*P* < 0.01) and T2 family relationships (*P* < 0.01), therefore, variance non-uniformity of data was used. In the *t*-test for equivalence of means, there was no significant difference in gender for social support and family relationships measured at the 2 time points (*P* > 0.05), whereas there was a significant difference in physical activity behaviors at T1 (*P* < 0.05), and therefore gender needs to be considered in subsequent model comparisons.

**Table 2:** Independent samples *t*-test for gender and Hukou location for T1 and T2.

**Grouping Variables**	**Implicit variable**	**HV-test**	**Levine's test of variance equivalence**		**Equality of means** ***t-*****test**	
			**F**	**P**	**t**	**P**
Distinguishing between the sexes	T1 Social support	Variance non-chirality	11.085	0.001^**^	−0.02	0.984
	T2 Social support	Variance chi-square	0.026	0.872	0.052	0.958
	T1 Physical Exercise behavior	Variance chi-square	8.804	0.003	−0.134	0.893
	T2 Physical Exercise behavior	Variance chi-square	13.369	< 0.01^**^	1.236	0.217
	T1Family Relationships	Variance chi-square	10.082	0.002	2.219	0.027^*^
	T2Family Relationships	Variance chi-square	6.715	0.01	0.226	0.821

“^*^” indicates *p* < 0.05, “^**^” indicates *p* < 0.01.

At the same time, an ANOVA was conducted to compare the age and academic level of the variables measured (as shown in Table 3), and it was found that there was no significant difference between the two-time points in terms of academic level and age (*p* > 0.05).

**Table 3:** ANOVA for grade and age for T1 and T2.

**Grouping Variables**	**Implicit variable**	**Mean square**	**F**	**P**
Grade	T1 Social support	0.421	1.351	0.25
	T2 Social support	0.069	0.202	0.937
	T1 Physical Exercise behavior	0.457	1.175	0.321
	T2 Physical Exercise behavior	0.532	1.46	0.214
	T1 Family Relationships	0.575	1.344	0.253
	T2 Family Relationships	0.563	1.209	0.306
Age	T1 Social support	0.138	0.438	0.781
	T2 Social support	0.159	0.471	0.757
	T1 Physical Exercise behavior	0.366	0.939	0.441
	T2 Physical Exercise behavior	0.123	0.333	0.856
	T1Family Relationships	0.541	1.264	0.283
	T2Family Relationships	0.233	0.498	0.738

### 4.4 Cross-lagged processes and analysis of social support, physical activity behavior, and family relationships among university students

**Figure 2:**
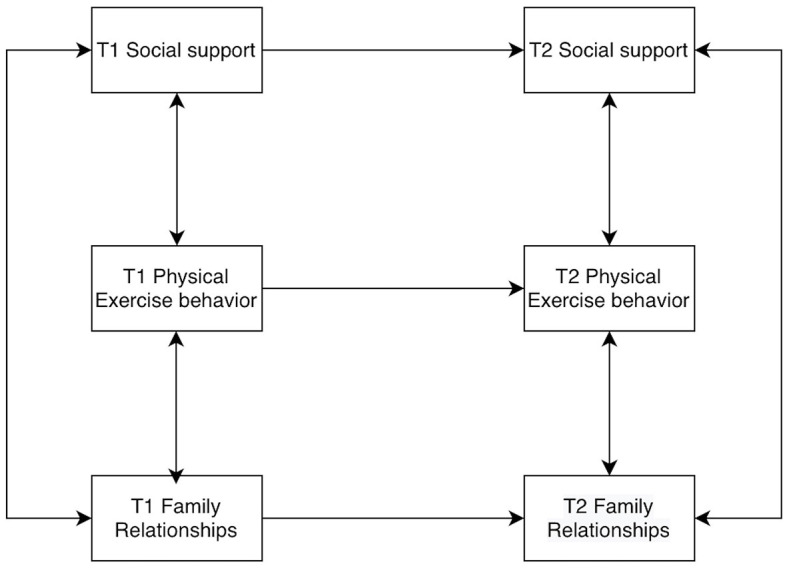
Baseline model M1.

**Figure 3:**
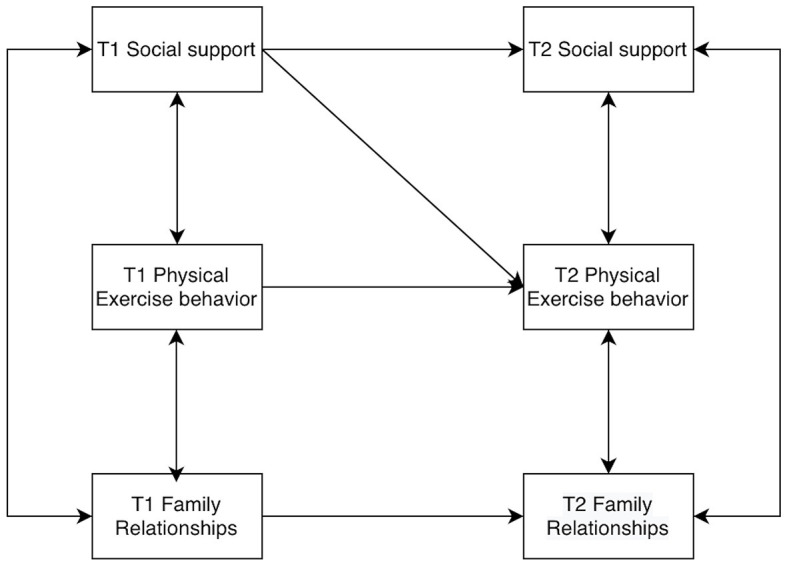
Model M2.

**Figure 4:**
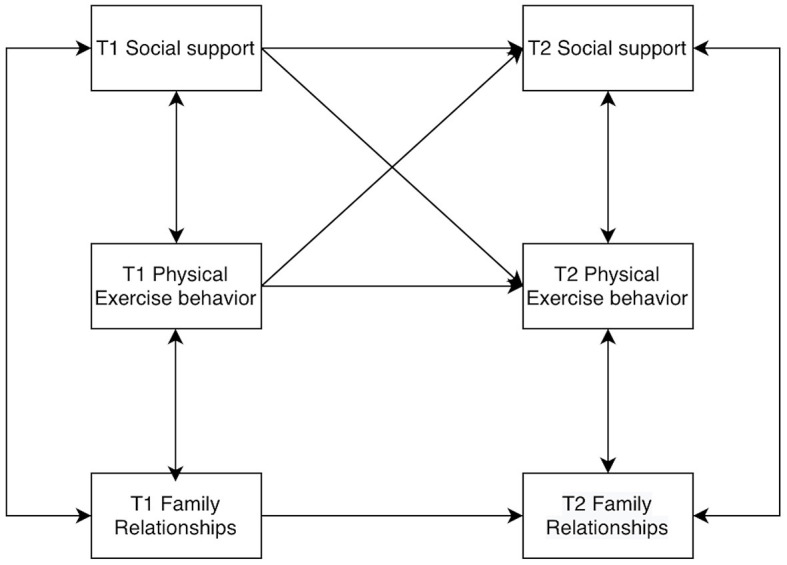
Model M3.

**Figure 5:**
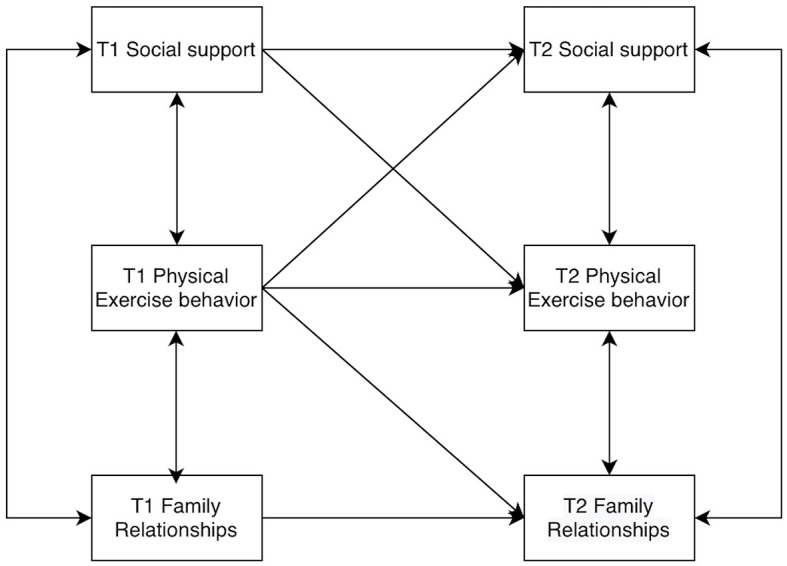
Model M4.

**Figure 6:**
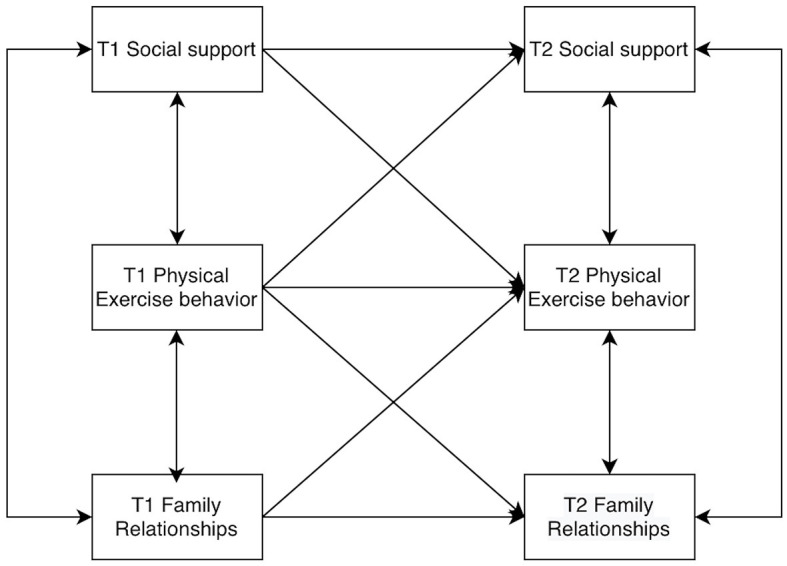
Model M5.

**Figure 7:**
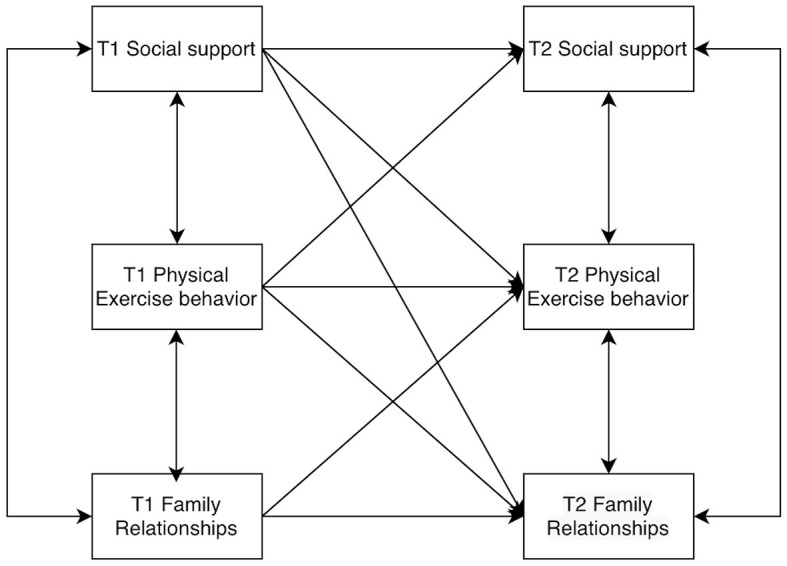
Model M6.

**Figure 8:**
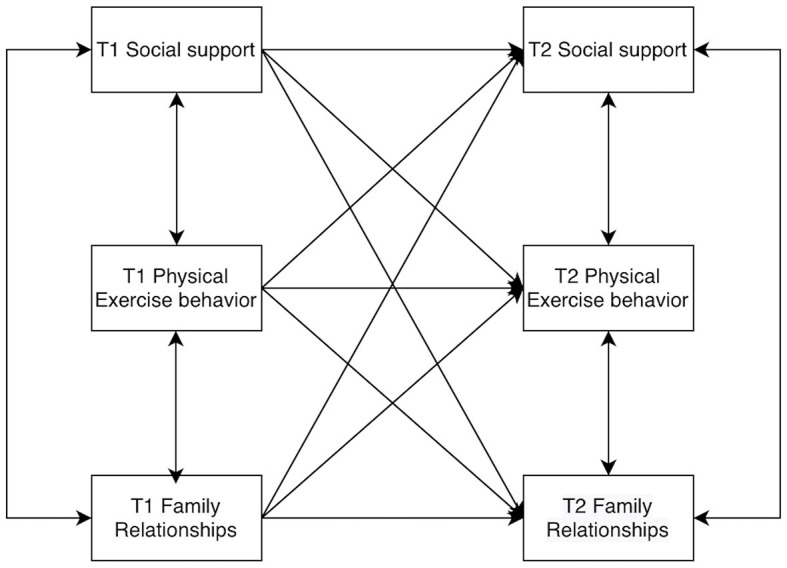
Full model M7.

**Figure 9:**
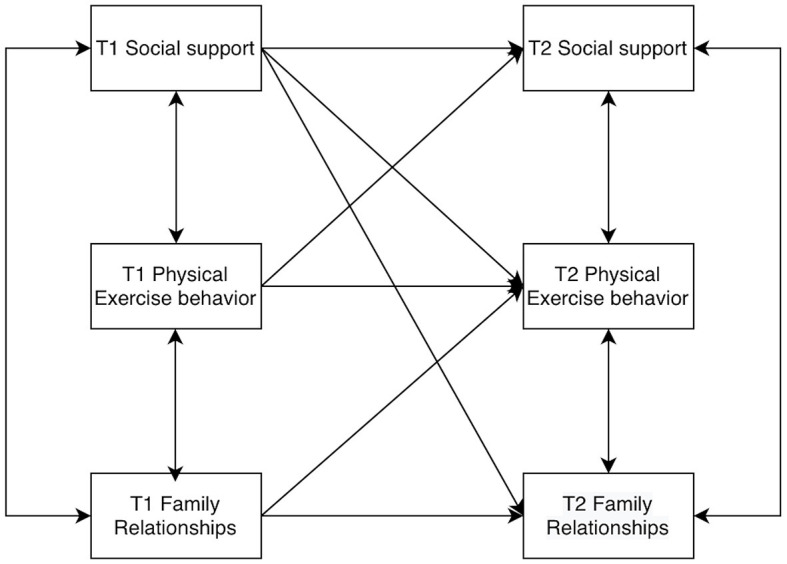
Final model M8.

Models of college students' social support, physical activity behaviors, and family relationships were constructed by AMOS 26.0, allowing for residual correlations at the same time points. The baseline model was established first (Figure 2), and then the M2 to M8 models were constructed according to the cross-correlations and paths (Figures 3–9). The path addition and fit metrics for each model are as follows (Table 4).

**Table 4:** Indicators of fit for models M1-M8.

**Model name**	**Add Path**	**χ^2^/*df***	**GFI**	**CFI**	**RMSEA**
M1	Baseline model	28.287	0.858	0.821	0.258
M2	X1 → Y2	28.490	0.874	0.840	0.259
M3	Y1 → X2	32.325	0.875	0.840	0.276
M4	Y1 → Z2	17.729	0.929	0.927	0.202
M5	Z1 → Y2	21.273	0.929	0.926	0.222
M6	X1 → Z2	2.815	0.991	0.995	0.066
M7	Full model	1.119	0.997	1.000	0.017
M8	Remove insignificant paths	1.294	0.995	0.999	0.027

X, Y, and Z represent social support, physical activity behavior, and family relationships, respectively, with the corner marker 1 representing the T1 time point and 2 representing the T2 time point. The process of adding cross-lagged paths is such that the latter model is added on top of the former model, e.g., M2 is added on top of M1 with path X1 → Y2.

**Figure 10:**
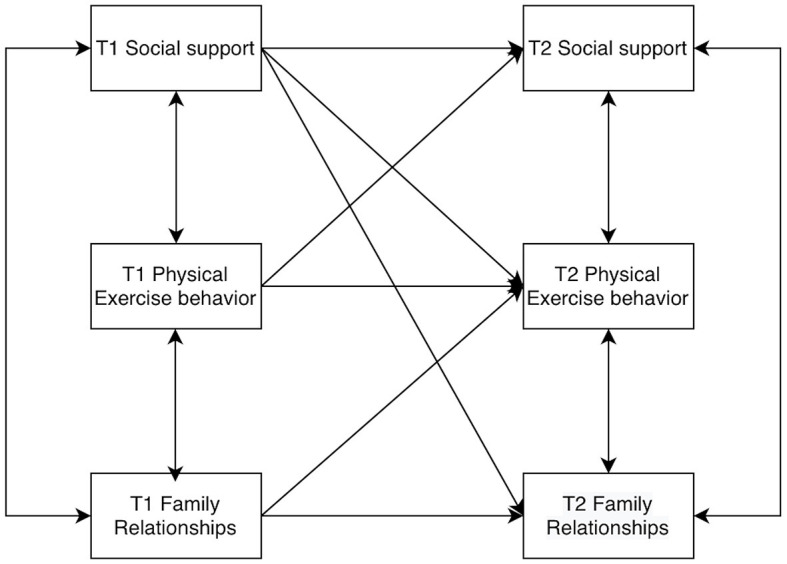
Final model M8.

**Figure 11:**
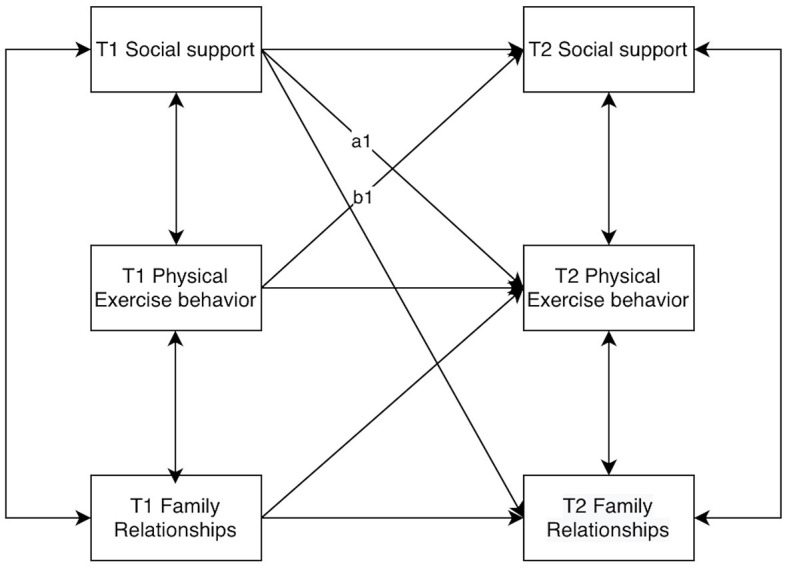
Equivalent model M9.

Model comparison was conducted through AMOS26.0, and it was found that among models M1 to M8, the fit of models M1 to M5 was unsatisfactory, and the fit indicators of models M6 to M8 were satisfactory, in which the 2 cross-lagged paths in model M7, T1 Physical Activity Behavior → T2 Family Relationships, and T1 Family Relationships → T2 Social Support were not non-significant, so model M7 was deleted and model M8 was selected. To further compare the fit between the equivalent model M9 (Figure 10) and M8 (Figure 11), the cross-lagged path equivalence setting was carried out, and the two cross-lagged paths T1 social support → T2 physical activity behavior and T1 physical activity behavior → T2 social support was set to be a1 and b1, respectively, and it was found that after setting that all the indexes of the fit of the model M9 deteriorated, and there was a significant M8 and M9 difference (*p* < 0.001) (Table 5), therefore, this equilibrium restriction is not reasonable, and the final model in this study is the cross-lagged model M8 (e.g., Figures 9, 11).

**Table 5:** Comparison of indicators for models M8 and M9.

**Model name**	**χ^2^/*df***	**GFI**	**CFI**	**RMSEA**	
M8	1.294	0.995	0.999	0.027	*P* = 0.000^***^
M9	4.455	0.978	0.985	0.092		

“^*^” indicates *p* < 0.05, “^**^” indicates *p* < 0.01, “^***^” indicates *p* < 0.001.

**Figure 12:**
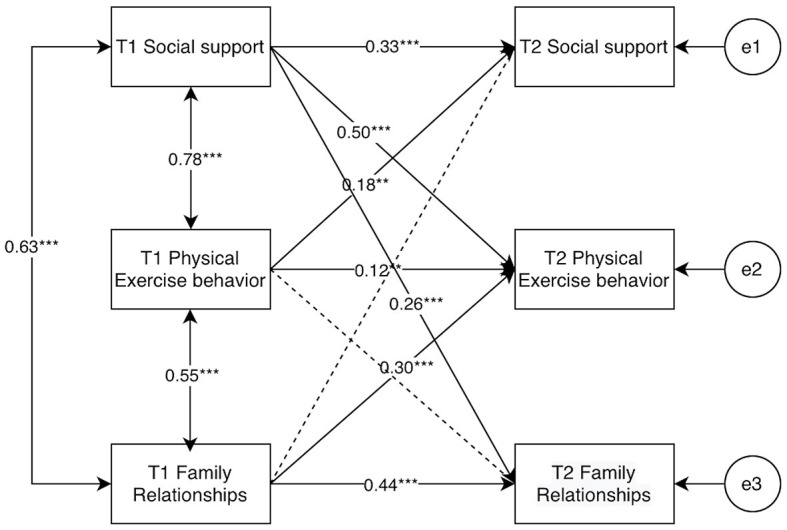
Results for each pathway for M8. “**” indicates *p* < 0.01, “***” indicates *p* < 0.001.

In the cross-lagged model of college students' social support, physical activity behavior, and family relationship (Figure 12), the various fit indices of the model were χ ^2^/*df* = 1.294, GFI = 0.995, CFI = 0.999, and RMSEA = 0.027, which indicated that the cross-lagged model of college students' social support, physical activity behavior and family relationship constructed in this study had a good fit. Bootstrap test analysis found that T1 social support was able to positively predict T2 physical activity behavior (β = 0.50, *p* < 0.001), and T1 physical activity behavior was also able to positively predict T2 social support (β = 0.18, *p* < 0.01), but the path value T1 social support → T2 physical activity behavior was greater than T1 physical activity behavior → T2 social support, therefore, the social support was a causal variable for physical activity behavior; T1 social support positively predicted T2 family relationships (β = 0.26, *p* < 0.001); and T1 family relationships positively predicted physical activity behavior (β = 0.30, *p* < 0.001).

**Figure 13:**
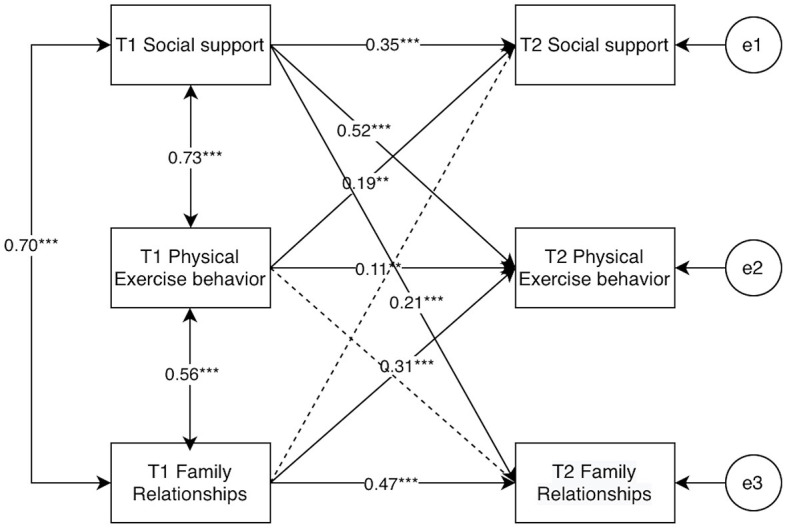
Results of each path for model M8 for male university students. “**” indicates *p* < 0.01, “***” indicates *p* < 0.001.

**Figure 14:**
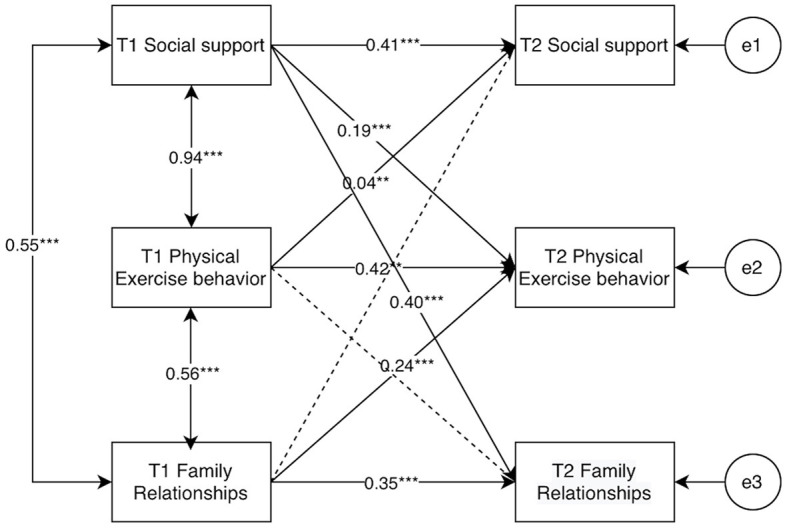
Results of each path for model M8 for female university students. “**” indicates *p* < 0.01, “***” indicates *p* < 0.001.

To further test the gender differences between college students' social support, physical activity behaviors, and family relationships, cross-group comparisons were set up in AMOS, Group1, and Group2 were established to represent male and female college students, respectively, male and female autoregressive and cross-lagged path equivalents were set up. The results of the running comparisons showed that there was a significant difference between unrestricted models and restricted models (χ^2^ = 241.29, Δ*df* = 16, *p* < 0.001), indicating that the cross-lagged models of social support, physical activity behavior and family relationships of college students differed by gender, as shown in Figures 13, 14.

After distinguishing gender, it was found that the cross-lagged paths of each cross-lagged path for female college students were larger than those of male college students, except for the path of T1 social support → T2 family relationship (β: 0.40 > 0.21); all other cross-lagged paths were smaller than those of male college students.

### 4.5 Analysis of vertical intermediation effects

According to the test of the amount of longitudinal mediating effects, the mediating effects of college students' family relationships and social support were analyzed across time (Li et al., 2022), and the Bootstrap method was applied to test the significance in AMOS26.0, with the sample size of 2,000 selected and the confidence interval set at 95%. Based on the cross-lagged model M8, T1 family relationship → T2 physical activity behavior was set as a1, T1 physical activity behavior → T2 social support as a2, T1 social support → T2 family relationship as c1, and T1 social support → T2 physical activity behavior as d1; the path coefficient of T1 family relationship → T2 physical activity behavior was 0.301, and the confidence interval was [0.173, 0.454], the path coefficient of T1 physical exercise behavior → T2 social support was 0.179, with a confidence interval of [0.014,0.336], and the longitudinal mediation effect size a1^*^a2 = 0.054, with a confidence interval of [0.009,0.121], *p* < 0.05, indicating that the longitudinal mediation effect was significant.

## 5 Discussion

This study reveals the longitudinal relationship between college students' social support, physical activity behaviors, and family relationships, as well as the mediating effect of physical activity behaviors in social support and family relationships, and it is a positive exploration of preventing and promoting the development of college students' physical and mental health. At the theoretical level, this study enriches the research on the factors and mechanisms influencing physical activity behavior and mental health and deepens the research results of physical education; at the practical level, it reveals the importance of physical exercise behavior, proves the intrinsic mechanism between college students' social support, physical activity behavior and family relationship, and provides a New Ideas.

### 5.1 The relationship between social support and physical activity behavior among university students

The findings of the current study indicate that college students' physical activity behavior significantly and positively predicts social support, thereby supporting hypothesis 1a. This is consistent with other researchers (Chen, 2001; Smith et al., 2017; Xu and Li, 2017). As mentioned earlier, physical activity behavior and social support both share the common effect that social support can reduce the probability of depression (Qiu et al., 2021), and good physical activity behavior can also cultivate the participants' emotions, morality, and character, which is a unique vehicle to develop socio-emotional competence and reduce the risk of depression (Zhao et al., 2024). The study further pointed out that college students' social support is a mediating variable between physical activity behavior and depression, in other words, physical activity behavior can improve depression by influencing social support (Zhang et al., 2022), which indicates that there is a correlation between physical activity behavior and social support. Therefore, college students' physical activity behavior can positively predict social support.

Also, college students' social support positively predicted physical activity behavior, and Hypothesis 1b was supported. College is the transition stage between adolescence and adulthood, and the formation and development of physical activity behavior at this stage is very important (Ji et al., 2022). At present, the lack of physical exercise among college students in China leads to poor physical fitness (Wang et al., 2020), which requires colleges and universities, and education departments to encourage and support college students to engage in physical exercise from the perspective of influencing factors, thus laying a good foundation for the development of lifelong sports. Some studies have found that the formation and development of college students' physical activity behavior are influenced by various aspects such as family, peers, society, academic pressure, and gender (Pan et al., 2022), among which, social, family, and peer support influence college students' physical activity behaviors (Bandura, 2004; Beets et al., 2010; Rackow et al., 2014). Therefore, college students' social support can positively predict physical activity behavior.

### 5.2 The relationship between family relationships and physical activity behavior among university students

In this study, college students' family relationships positively predicted physical activity behavior, and Hypothesis 2a was established. This is in line with the views of previous researchers (Thompson and Meyer, 2013; Wang, 2019; Liu, 2021; Pan, 2022). Physical activity behavior is influenced by many aspects, including family, individual, and society (Pan et al., 2022). The family unit is the primary catalyst for an individual's growth and the foundation for the development of physical activity behaviors. Family systems theory posits that parents play a pivotal role in their children's maturation (Bowen, 1966), which is manifested in the fact that the support given to children by family members (e.g., parents) improves the individual's physical fitness (Tandon et al., 2012; Dong et al., 2018), so families should give individuals the opportunity to participate in physical exercise support, thus promoting the overall development of individuals and laying a good foundation for lifelong sports. Concurrently, positive familial dynamics can facilitate children's development of the three essential perspectives (Calatrava et al., 2023). This approach also serves to establish the correct values of sports and improve the level of individual physical activity behavior. Therefore, college students' family relationships can positively predict physical activity behavior.

In addition, college students' physical activity behaviors do not predict family relationships, Hypothesis 2b holds. Research in the current field focuses on the relationship between family sports and individual sports participation (Pan, 2022; Liu et al., 2023). Although research proposes that family physical activity can influence family relationships through intergenerational transmission effects (Wang et al., 2016), the current situation of college students in China is manifested by the fact that students are mainly boarding on-campus (He and Yang, 2014), and participation in physical activity with family members during the school year is limited by time and space. At the same time, as mentioned earlier, improving physical health, recreation, and weight loss are the motives for college students to participate in physical activity during school (Li et al., 2022), and there is no strong correlation with the development of family relationships. Therefore, college students' physical activity in school does not significantly affect family intimacy (family relationships), i.e., college students' physical activity behavior does not directly predict family relationships.

### 5.3 The relationship between family relationships and social support for college students

The results of the current study indicated a significant positive correlation between social support and family relationships at the same time point. However, cross-lagging analysis revealed that college students' family relationships did not predict social support, and Hypothesis 3a was not supported. Additionally, T1 family relationships did not predict T2 social support across time, which differed from previous studies (Zhao et al., 2011; He et al., 2018; Wei et al., 2021). Previous studies have only explored the link between family relationships and social support individually and in cross-sectional studies. Meanwhile, the individual's ability to appreciate social support is affected by the individual's social experience (Hartley and Coffee, 2022; Ma et al., 2023), college students have less social experience and make little use of social support (Liu, 1998), and in the group of college students, social support is affected by many aspects such as individual psychology, school, society, and family, etc. Due to the special nature of college students' living environment, they tend to be in on-campus accommodation during their school years (He and Yang, 2014), and the connection with family members is reduced, which is also an objective reason why family relationships of college students cannot predict social support. Therefore, this study introduces another variable: physical activity behavior, aiming to deeply analyze the correlation mechanism between college students' social support, physical activity behavior, and family relationships. The fact that family relationships do not directly predict social support suggests that there may be a deeper longitudinal relationship between social support, physical activity behavior, and family relationships.

Meanwhile, the results of the current study indicate that college students' social support can positively predict family relationships, and Hypothesis 3b is supported. This is consistent with previous studies (Gruijters, 2017; Wei et al., 2021). On the one hand, social support can alleviate individuals' negative emotions and negativity in life, which in turn improves psychological wellbeing (Tabatabaei et al., 2018; Cohen and McKay, 2020), and individuals who maintain a pleasant mood are more willing to communicate with their family members, which in turn promotes the development of family relationships. Social exchange theory suggests that the acquisition of social support must be maintained in a balanced view with the provision of support (Ahmad et al., 2023), meaning that people can obtain support from their social relationships and need to provide support for others (Clark et al., 1987). Family is the constituent unit of society, and social support includes mutual support among family members, while individuals are supported by their family members, they also need to provide support for their family members, which promotes the development of family relationships in the process of mutual support. Therefore, social support for college students can positively predict family relationships.

### 5.4 An analysis of the underlying mechanisms of social support, physical activity behavior, and family relationships among university students

Despite the lack of predictive power of family relationships on social support in the present investigation and the absence of a direct temporal predictive link between physical activity behavior and family relationships, owing to the unique nature of the academic environment encountered by university students, the present study analyzed the mediating effects among social support, physical activity behavior, and family relationships among this cohort, drawing upon the outcomes of the cross-lagged analysis. It was found that in the college student population, physical activity behavior was the mediating effector between family relationships and social support, and Hypothesis 4 was supported. This demonstrated that although college students' family relationships cannot directly influence social support, they can further have an impact on social support through physical activity behaviors. Perfection theory suggests that family relationships can influence the behavior of individuals (Liu, 2021), whereas family support in family relationships can influence students' physical activity behavior (Davison et al., 2004). Similarly, some studies have shown that the higher the frequency of participation in physical activity, the greater the subjective support and support utilization that can be obtained (Xu and Li, 2017), and physical activity behavior shows a significant positive correlation with social support (Sun et al., 2023). As further mentioned in previous studies, family relationships (e.g., family closeness and adaptability) are associated with social support, and family relationships can indirectly influence social support (Luo and Zhang, 2015). This indicates that physical activity behavior can act as a mediating variable between family relationships and social support. In other words, the family relationships of college students must influence the availability of social support through physical activity behavior.

### 5.5 Strengths of the study and future directions

This study explored the interrelationships between college students' social support, physical activity behaviors, and family through a cross-lagged design. First, this study longitudinally tracked and collected two rounds of data from the college student population with a 2-month interval to provide a more detailed analysis of this phase of the college student population. Second, we discussed the relationship between different variables by gender, which is conducive to understanding the unique roles of these variables across genders. Finally, in the design of the methodology, the differences were first compared by independent samples *t*-test and ANOVA, then the best model was selected by comparing the fit of the model, and the differences between the male and female gender models were compared based on the best model, which resulted in a more robust methodological design and a more in-depth analysis of the data.

## 6 Conclusion

The cross-lagged study led to the conclusions of this paper that (1) there are gender differences in college students' social support, physical activity behaviors, and family relationships; (2) college students' social support is a causal variable for physical activity behaviors; (3) social support positively predicts family relationships; (4) family relationships positively predict physical activity behaviors; and (5) physical activity behaviors are a mediator variable between family relationships and social support.

Therefore, this study suggests that colleges and universities should pay attention to college students' school communication and family relationships, and through regular psychological lectures, let college students learn how to communicate with their relatives and friends, reduce the occurrence of family conflicts and friendship conflicts among college students, and encourage college students to actively participate in sports, through participation in sports activities, college students can make new friends, expand their social circle, and enhance their interpersonal communication skills and teamwork.

In summary, this study explored the mechanism of college students' social support, physical activity behavior, and family relationship through longitudinal tracking survey and cross-lagged model design and analysis, but it still needs to be paid attention to and improved in future research: (1) the tracking length of the study can be further extended, for example, future researchers can conduct 2–3 surveys through half a year or 1 year; (2) in addition to exploring the mechanism underlying the social support and physical activity behavior, social support and family relationship, and the intrinsic mechanism of physical activity behavior and family relationship, there is still a need to pay attention to and consider the possible roles and influences of other factors on the social support, physical activity behavior and family relationship of college students, and there is a need to further consider other variables in the future for research.

## Data availability statement

The original contributions presented in the study are included in the article/Supplementary material, further inquiries can be directed to the corresponding author.

## Ethics statement

The studies involving humans were approved by the Ethics Review Committee of Chengdu Sport University Research Office. The studies were conducted in accordance with the local legislation and institutional requirements. The participants provided their written informed consent to participate in this study.

## Author contributions

XZ: Conceptualization, Data curation, Formal analysis, Investigation, Methodology, Software, Validation, Writing – original draft, Writing – review & editing, Supervision. MZ: Supervision, Validation, Writing – review & editing. BL: Conceptualization, Supervision, Validation, Writing – review & editing. SM: Supervision, Writing – review & editing.

## Footnotes

^1^China Youth Daily. *Sports survey of 25,000 college students finds: nearly 50 per cent exercise less than three times a week [EB-OL]*. Available at: https://baijiahao.baidu.com/s?id=1774790002103436544&wfr=spider&for=pc.
